# Supplementary material for: Are carbon emissions trading and green financial instruments synergistic? -Comprehensive quantitative research based on content analysis
Source: PLoS One. 2024 Mar 7;19(3):e0298601. doi: 10.1371/journal.pone.0298601 (PMC10919601; doi:10.1371/journal.pone.0298601)
Supplement: S1 Data — (ZIP) [file pone.0298601.s001.zip › Data/地级市Z_F工作报告.zip!/╣≤╓▌/╣≤╤⌠╩╨/└┤╫╘╣1⁄2╓┌║┼í╛┬φ┐╦╩2╛▌═°í┐_2007.docx]

各位代表：

本届政府2002年12月就职，任期即将结束。现在，我代表市人民政府向大会作工作报告，请予审议，并请各位政协委员和列席会议的同志提出意见。

一、本届政府工作回顾和2006年经济社会发展情况

市十一届人大一次会议以来，我们在省委、省政府和市委的正确领导下，在市人大及其常委会、市政协的监督、支持下，坚持以邓小平理论和“三个代表”重要思想为指导，全面落实科学发展观，着力构建社会主义和谐社会，紧紧依靠全市人民，认真贯彻执行中央和省一系列决策部署，在排除各种困难和风险中开拓前进，顺利完成“十五”计划，圆满完成了市十一届人民代表大会历次会议确定的各项目标任务，改革开放、经济建设、社会发展取得巨大成就，全市呈现出蓬勃发展、文明进步、安定和谐的崭新局面。

（一）经济持续快速发展，综合实力跃上新台阶

全市生产总值从2002年的348.02亿元增加到2006年的602.88亿元，按可比价格计算，年均增长14.1%，实现了市十一届人大一次会议提出的比2000年翻一番的目标。人均GDP从10123元增加到17025元。财政总收入从79.11亿元增加到157.58亿元，年均增长18.8%。地方财政收入从33.08亿元增加到77.15亿元（地方所得税按100%口径），年均增长23.6%。固定资产投资从187.96亿元增加到413.11亿元，年均增长21.7%。城市居民人均可支配收入从7306元提高到11222元，年均实际增长10%；农民人均纯收入从2352元提高到3465元，年均实际增长7.8%。

（二）结构调整成效显著，经济自主增长能力明显增强

三次产业结构由2002年的7.7：47：45.3调整为2006年的6.3：48.4：45.3，非公有制经济比重逐年增加。工业经济结构进一步优化，磷化工、铝及铝加工、煤化工、装备制造、卷烟、能源等现实优势产业对全市经济的支撑作用进一步增强，以新材料、电子信息产品、现代中药业为代表的一批高新技术产业快速发展。全市有年销售收入在50亿元以上企业2个；中国名牌产品从无到有，贵州轮胎公司“前进”牌全钢子午线轮胎、“老干妈”油制辣椒等4个产品荣获中国名牌产品称号，产业集群化、工业多点支撑的局面初步形成。大力发展都市农业、城郊农业、特色农业、高效农业、生态农业，加快农业产业化发展步伐，八大优质农产品产业带初步形成，蔬菜、花卉、畜牧业等产业成为农业新的经济增长点。通信、旅游、信息、房地产等服务业渐成规模，第三产业在国民经济中的比重不断提升。

（三）基础设施建设步伐加快，城市集聚力、辐射力、带动力显著提高

强力推进“三轮驱动”战略，努力加大交通、水利、信息、环保和城市公用设施等基础设施建设力度，城市发展基础和环境得到较大改善。四年累计完成基本建设投资562.5亿元。建设、改造县乡公路2400公里、村公路2358公里，完成串户路硬化1800公里，2003年在全省率先实现乡乡通油路，2006年在全省率先实现县县通高等级公路，市域一小时交通网络已经形成。治理病险水库64座，率先在全省阶段性消灭病险水库，完成鱼简河水库、花溪水库扩建加高等水利工程，解决农村38万人饮水困难，提高了城市防洪和供水能力。实施退耕还林、天然林资源保护、“二环林带”等生态建设工程，全市森林覆盖率达34.77%。建立起较为完善的光纤宽带城域网和现代远程教育网，贵阳地理信息系统一期工程全面完成。新建、改扩建37条城市道路和一批城市自来水厂、污水处理厂、垃圾处置场，城市绿化覆盖率达40.47%，城市供水设计能力达100万吨/日，空气质量优良天数占全年的93.6%。实施“南明河三年变清”系列工程，实现“水变清、岸变绿、景变美”的阶段性目标，获得“中国人居环境范例奖”和“水环境治理优秀范例城市”称号。金阳新区建设实现突破，提前4年完成17平方公里范围内基础设施建设，基本完成市级行政中心一、二期工程、学校、医院、公园、住宅等一批城市功能配套设施，房地产开发有序推进。由卫星城、重点镇、一般镇构成的市域小城镇体系基本形成，小城镇道路铺装率达90%，镇区供水率达100%，基本实现了国（省）道沿线小城镇场路分开。荣获“国家森林城市”、“全国绿化模范城市”、“中国十佳宜居城市”、“中国避暑之都”等称号。

（四）改革开放取得重大进展，经济发展活力、动力持续增强

国有资产管理体制进一步完善，全面启动国有企业改革重组，完成国企改革重组71户，分离企业办学校32所，国有大中型企业历史包袱明显减轻，国有资产监管体制进一步完善。完成农村税费改革试点和农村信用社改革，启动农村综合改革试点。投融资体制改革取得重大进展，投资主体多元化的格局基本形成，企业投资主体地位进一步巩固，政府投资行为进一步规范，全市固定资产投资从2002年的187.96亿元增加到2006年的413.11亿元，年均增长21.7%。行政审批体制改革不断深入，市政府规章自行设定的77项行政许可全部取消。以部门预算管理、财政国库管理和政府采购制度为重点的财政体制改革取得新的成绩。外贸进出口保持强劲增长，全市外贸进出口总额从2002年的5.7亿美元增加到2006年的11.19亿美元，年均增长18.2%，全市出口商品近800种，与我市开展贸易往来的国家和地区达100多个。招商引资成效显著，全市实际直接利用外资从2002年的2918万美元增加到7010万美元，年均增长24.5%。

（五）社会事业全面进步，和谐贵阳魅力彰显

大力实施“科教兴市”战略，累计投入科技三项费2.34亿元，实施市级科技项目681个，连续三届获得全国科技进步先进市称号，成为全国制造业信息化示范城市、国家知识产权试点城市。教育、文化、卫生、计生事业取得长足进步，贵阳一中新校、贵阳大剧院、贵阳市美术馆等建成并投入使用，贵阳学院完成组建，率先在全省实现“两基”目标，全市人均受教育年限上升到8.8年，人口自然增长率从8.17‰下降到5.67‰。多渠道筹集资金改善文化基础设施，四年累计投入资金5.6亿元用于文化基础设施建设。依法治市、依法行政取得显著进展。共办理人大代表建议和政协提案2175件，提请市人大常委会审议通过地方性法规24件，制定并公布市政府规章50件。加强行政监察和审计监督，廉政建设取得新成绩。持久开展社会治安综合治理，连续三次被评为“全国社会治安综合治理优秀城市”并获得“长安杯”。安全生产形势总体趋于平稳。公安、司法、统计、物价、工商、质监、新闻出版、民政、民族、宗教、人防、社会科学、外事侨务、对台、市志、档案等各项事业取得明显进步。

（六）人民生活不断改善，全社会凝聚力有效提升

城市居民人均可支配收入、农民人均纯收入分别由7306元、2352元增加到11222元、3465元，年均分别增长10%和7.8%。城乡居民住房条件得到改善，城市居民和农村居民人均住房面积分别由14.91平方米、26.25平方米增加到16.7平方米、35.5平方米。实施农村茅草房改造工程，使37562户、15万农民告别了世代居住的茅草房。整村推进、产业化扶贫和贫困地区劳动力转移三项重点工作取得明显成效。就业形势基本稳定，累计实现城乡统筹就业30.8万人，城镇登记失业率控制在4.1%以内。建立了基本养老、基本医疗、失业、工伤、生育五险齐全的社会保险制度，“两个确保”100%的成果进一步巩固，“三条保障线”政策全面落实，在实现城市低保“应保尽保”和“分类施保”的基础上再次提高了低保金标准，农村低保全面铺开，率先在全省全面推行新型农村合作医疗和开展城市医疗救助试点工作，把农民工纳入基本医疗保障。坚持每年为人民群众办十件实事，累计投入资金78.5亿元，完成实事项目40大项152个子项，惠及310万人民群众。

各位代表，在刚刚过去的2006年，我们认真贯彻市十一届人大五次会议的决议要求，开拓奋进，狠抓落实，经济社会发展呈现出近年少有的发展势头。初步核算，全年生产总值达到602.88亿元，比上年增长14.7%，这是多年来增幅最高的一年。第一产业增加值增长7.6%；第二产业增加值增长16.7%；第三产业增加值增长13.6%。财政总收入、地方财政收入、固定资产投资、全社会消费品零售总额、城市居民人均可支配收入、农民人均纯收入分别比上年增长18.4%、23.7%、20.1%、15%、13%、10.5%。

——突出结构优化升级，工业经济整体质量明显提高。重点行业竞争能力有较大提高，烟草制品、有色金属冶炼及压延加工、医药制造、化学原料及化学制品、电力生产和供应、黑色金属冶炼及压延加工、交通运输设备制造、橡胶制品、通信设备计算机及其他电子设备制造等九大支柱产业占全市规模以上工业增加值的80％以上。高技术产业占全市规模以上工业增加值的比重达16.4%，聚合物锂电池、中央空调节能器等产品获国家重点新产品奖。非公有制经济发展迅速，非公有制经济增加值比上年增长16%，占全市生产总值的比重达42%。清洁生产、资源节约与综合利用工作深入开展，工业固体废弃物和黄磷尾气综合利用、公交车油改气、沼气池建设等循环经济项目顺利推进。实施了50户国有企业改革改制工作。

——突出重点项目带动，固定资产投资稳定增长。全市纳入跟踪的109个重点项目建设达到预期目标。开磷集团120万吨磷铵、清镇配煤基地45万吨/年煤矿等重点项目开工建设，中化开磷有限公司60万吨多功能复合肥、贵州信邦远东制药有限公司GMP改造等项目建成投产，贵州轮胎公司全钢子午线轮胎、贵阳海信数字平板电视生产线等项目取得较好效益。1000公里农村等级公路全面完成，贵开二级公路竣工通车、绕城高速公路、贵阳铁路枢纽等一批基础设施项目进展顺利。基本完成138项财政性投资项目“投转固”工作。

——突出生产发展，社会主义新农村建设开局良好。深入开展“十村示范、百村先行、千村推进”工程，完成100个行政村的新农村建设规划。支农力度进一步加大，全市整合各类资金10多亿元投入到新农村建设中。坚持“水、电、路、气、房、林、电视、电话”十字方针，农村生产生活设施明显改善，荣获“中国十大政府创新典型”奖。772公里串户路硬化建设、7万口“四改一气”沼气池、农村5.09万人饮水安全工程、农田水利基本建设全面完工，阳朗排洪工程全面完成。1597个自然村通电视，845个自然村通广播，农村现代远程教育覆盖六区三县一市和80个乡镇。农业产业结构调整取得新突破，蔬菜总产量突破百万吨，完成花卉大棚建设2000个，启动养殖小区建设项目318个。命名28家市级龙头企业，扶持农业专业合作经济组织20个。完成农村富余劳动力非农技能培训2.67万人，有序转移2.18万人。

——突出规模扩大和层次提升，第三产业加快发展。消费品市场活跃，商贸流通、交通运输等传统行业保持较快发展，住房、视听产品、游戏娱乐用品等仍是消费热点。市场建设成效显著，浙江商城一期工程、西南国际家居装饰博览城等大型批发市场建成开业，中天花园等3个绿色农贸市场建成，“万村千乡”市场工程顺利推进，完成农家店改造220个。全市社会消费品零售总额完成234.96亿元，同比增长15%。以荣获“中国避暑之都”称号为契机，加大旅游宣传推介和拓展市场力度，全市旅游业呈现良好的发展势头。全年共接待国内游客1844.75万人次，增长27%；接待海外游客8.92万人，旅游创汇3904.16万美元，旅游总收入84.54亿元。

——突出开放带动，进一步拓展发展空间。成功举办和组织参加贵阳投资环境（杭州、广州）推介会、中国（贵阳）信息技术博览会、贵阳市台资企业协会成立暨制造业推介会、黄果树旅游节贵阳投洽会等9次大型招商活动。积极推进贵阳城市经济圈建设，加强与省内地州市及国内外大企业的交流与合作，拓展了对外合作的领域和空间。引进美国微软、法国斯奈克玛、法国拉法基、泰国正大等四家世界五百强企业，引进投资额5000万元以上大项目达101个。全市进出口总额完成11.19亿美元。全年共引进内资项目1626个，实际到位资金209.64亿元，同比增长30.2%；批准外商投资项目30个，实际直接利用外资7010万美元，同比增长16%。

——突出功能完善和形象提升，城市建设和管理取得新成效。完成《贵阳市土地利用总体规划修编大纲》并通过评审，启动贵阳城市总体规划修编（2006—2020年）工作。完成贵阳城市轻轨、奥林匹克体育中心、金阳“文化山”等一批事关长远的重大项目的规划、设计、评审工作。35个年度重点市政基础设施项目全面完工，贵惠路、四方河联络线等7条城市道路以及太平路、贵阳六中等9个人行过街系统工程竣工并投入使用，小河污水处理厂二期等3个环境整治项目顺利推进。金阳新区建设取得质的飞跃和量的突破，市级行政中心二期、贵阳三中新校等功能性设施建成并投入使用，新世界中国地产、世纪金源地产、贵金线、金元国际新城等一批建设项目有序推进。继续实施“为民办实事，满意到万家”和“整脏治乱”活动，大力推行精细化、人性化管理，城市环境绿化、美化、亮化、净化水平进一步提高，荣获省级文明城市称号。新增停车位3214个，面积42079平方米，建成16座公厕、8座垃圾转运站和52座环卫工人休息室，拆除不规范户外广告牌匾15584块，拆除违法建筑33万平方米。完成市中心区“城中村”整治规划方案。

——突出协调发展，构建和谐社会取得新进展。科技工作成绩突出，实施科技项目243个，成果鉴定75个。教育事业取得新的进步，出台了全市义务教育阶段办学标准，农村义务教育全部实现以县为主的管理体制，对5个区和18个贫困乡（镇）农村学校义务教育阶段的学生实行了“两免一补”，对城市低保家庭子女实行了“两免”，公办中小学接收农民工子女就读人数达1.6万人，高中阶段教育入学率达62%，省级示范高中达13所，占全省的38%。卫生事业再上新台阶，全面实施了新型农村合作医疗制度，艾滋病、结核病等重大疾病和人畜（禽）共患疾病得到有效控制。文化体育事业健康发展，贵阳大剧院投入使用，现代京剧《盘江屋里人》初选进入国家舞台艺术精品工程，市第十届全运会暨第四届职工运动会、白云风筝节等大型活动成功举行，实施了有线电视“模转数”工程。安全生产工作在全省名列前茅，安全事故发生起数、事故死亡人数均比上年同期大幅下降。社会治安综合治理工作成效显著。劳动就业规模继续扩大，社会保障体系不断完善，实现城乡统筹就业7.1万人，城镇登记失业率为4%。

各位代表，本届政府履职以来，国民经济和社会实现了又好又快的发展，这是省委、省政府以及市委正确领导和亲切关怀，人大、政协支持的结果；是全市各族干部群众抢抓机遇、加快发展、奋力拼搏、团结奋斗的结果。在此，我代表市人民政府，向全市工人、农民、知识分子、干部，向各民主党派、工商联、无党派人士、人民团体，向驻筑人民解放军指战员、武警部队官兵、公安干警，向所有关心和支持我市改革开放和现代化建设的各界人士，表示衷心的感谢并致以崇高的敬意！

成绩来之不易，这主要得益于我们在工作中始终坚持以科学发展观为统领，用发展的办法解决前进中的问题，用市场经济的手段抓经济工作，用创新的精神培植发展新优势，抢抓发展机遇，加快发展步伐；始终抓住事关全市经济社会发展全局的战略问题，集中力量，攻破难关，办成了长期以来一些想办而没有办成的大事和实事；始终抓住结构调整这条主线，依托我市资源和特色优势，转变经济增长方式，着力提高经济增长的质量和效益；始终抓住改革开放这一根本动力，以改革促发展，以开放促开发，大力招商引资，借助外力加快自身发展；始终坚持以人为本，努力做到“民有所呼，我有所应，必有所为”，认真解决人民群众最关心、最直接、最现实的利益问题，努力改善社会管理、城市管理的方法和手段，让人民群众分享更多的改革成果和实惠，努力构建和谐贵阳。

各位代表，贵阳经济社会发展成果令人鼓舞，前进中也面临不少矛盾和问题：城市综合实力不强、经济总量不大、结构欠优，仍然是困扰我市经济社会发展的主要矛盾；城乡、区域发展不平衡，城乡二元结构矛盾仍较突出，统筹城乡发展的任务艰巨；城市基础设施还不够完善，人口与资源、环境容量矛盾突出，主城区交通拥堵等问题严重；体制障碍仍然较多，市场配置资源的基础性作用还未充分发挥，创业发展环境还需改善；市级财政进入偿债高峰期，平衡财政收支难度增大，调控能力减弱；影响和谐社会建设的因素仍然较多，许多群众关心的热点和难点问题亟待解决。这些事关全局的重大的问题，我们一定要高度重视，认真对待，努力解决。

二、今后五年政府工作的建议

各位代表，未来五年是我市加快现代化进程的重要时期。经过改革开放以来的积蓄发展，全市综合经济实力迈上新的台阶，经济社会发展站在新的起点，工业化、城镇化、现代化进入新的阶段。我们既面临产业结构加快调整、消费结构加快升级、城镇化步伐加快推进、社会结构加快转型、体制机制加快完善等发展机遇，也面对区域竞争日趋激烈、发展与资源生态矛盾尖锐、城乡协调发展任务艰巨、就业与社会保障压力仍较突出、各类社会矛盾凸显等方面的挑战。纵观国内外宏观环境变化趋向，总体上有利于实现又好又快发展。我们将按照中国共产党贵阳市第八次代表大会的统一部署，瞄准新的发展目标，立足新起点，增创新优势，实现新跨越，谱写贵阳历史的新篇章，铸就贵阳发展的新辉煌。

今后五年政府工作的指导思想是：坚持以邓小平理论和“三个代表”重要思想为指导，全面贯彻科学发展观，努力构建和谐社会，牢记胡锦涛总书记“做表率、走前列”的重托，围绕建设生态经济市的目标定位，突出更好更快发展的主题，立足新起点，增创新优势，实现新跨越。坚定不移地实施环境立市、工业强市、科教兴市、人才强市、可持续发展五大战略，调整经济结构，转变经济增长方式，深化改革，扩大开放，提高自主创新能力，全面加强经济建设、政治建设、文化建设和社会建设，加快推进社会主义新农村和实力贵阳、宜居贵阳、魅力贵阳、开放贵阳、创新贵阳、法治贵阳、和谐贵阳建设，率先在全省实现经济社会发展的历史性跨越。

今后五年贵阳经济社会发展的主要奋斗目标是：全面完成“十一五”规划，生产总值突破1000亿元大关，人均生产总值达到3000美元；全社会固定资产投资年均增长16%左右，累计比“十五”翻一番以上；单位地方生产总值能源消耗比“十五”期末降低20%；地方财政收入年均增长13%左右；社会消费品零售总额年均增长14%左右，累计增加城镇就业20万人以上，城镇登记失业率控制在4.5%以内；城镇化率提高到68%左右；城市居民人均可支配收入和农民人均纯收入均年均增长8%左右；人口自然增长率控制6‰以内。

要实现上述目标，我们必须按照科学发展观和构建和谐社会的要求来安排谋划一切工作，强化政府自身建设，努力在全省“做表率、走前列”。为此要着力抓好以下几方面的工作。

（一）以科学发展观为统领，在全省率先实现贵阳经济建设新跨越

1、扎实推进社会主义新农村建设，增创“三农”工作新优势。一是加快农业和农村经济结构调整。按照发展现代农业的定位和要求，大力实施农业标准化、规模化、产业化工程，大力发展农业产业化经营，扶持龙头企业，规划建设一批特色优质种养殖业区、农业标准化生产基地、科技示范基地、观光园区和生态养殖小区。加快中药材、辣椒、畜产品、果品等农产品加工型企业的发展，建设无公害优质农产品果树、蔬菜、中药材等八大优势产业带，打造农业知名品牌。加快实现农业机械化的进程，构建农机服务体系。二是加强农村基础设施建设。继续抓好以“水、电、路、气、房、林、电视、电话”为重点的农村基础设施建设。加快渔洞峡、席关水库等亚洲开发银行贷款水资源综合管理项目的建设，确保“十一五”期间解决农村60万人饮水安全问题。加强耕地特别是基本农田的保护，逐步改善农业的生产条件。全面完成农村电网改造，实现城乡用电同网同价。加快农村等级公路和村寨串户路建设，完成20户以上的村寨串户道路硬化。加快农村沼气池建设，全面完成农村18.5万口沼气池建设任务。加强农村广播电视网络、通讯网络和互联网络建设，发展农村现代远程教育，构建市、县、乡、村四级信息网络，实施农业综合信息服务平台建设，实现5户以上自然村寨全部通广播电视。三是加强农村公共服务体系建设。加大对农村的投入力度，使公共财政向农村倾斜，公共服务向农村延伸，公共管理向农村覆盖。加强农村公共卫生和基本医疗服务体系建设，完成乡镇卫生院改造、村卫生室建设及其基本设备配套，完善新型农村合作医疗制度，使农村初级卫生保健水平达到小康型合格县标准。加强农村文化建设，不断提高农村社会文明程度。四是加快农民转移就业步伐。鼓励发展农村二、三产业，加快农村劳动力向中心镇集聚和向非农产业转移。加强农业实用技术培训和非农技术培训，加大农村劳动力转移力度。五是加强新阶段扶贫开发的力度。继续采取整村推进、产业化扶贫和贫困地区劳动力转移等方式，使农村贫困人口实现脱贫或基本生活有保障，逐步建立抑制返贫的长效机制。认真落实移民政策，切实做好库区移民直补和项目扶持工作。

2、大力推进产业结构升级，增创经济建设新优势。一是大力推进“工业强市”战略。立足资源、能源优势和现有产业基础，加快发展高新技术产业，加大资金投入和政策扶持力度，用先进适用技术改造提升传统产业，培育壮大特色优势产业。加快形成磷化工、铝工业、现代中药、绿色食品、电子信息、装备制造六大产业集群，抓好一批工业重大项目建设，培育一批核心竞争力强的强势企业和一批具有自主知识产权、市场占有率高的名牌产品。争取形成若干个百亿元以上的产业集群，2个百亿元以上的大型企业或企业集团，培育一批年销售收入10亿元至50亿元的骨干企业。二是积极发展现代服务业。全力打造“森林之城、避暑之都、休闲胜地”的城市旅游形象。加快实施重点带动和品牌发展战略，进一步强化全省旅游业发展的龙头优势，提高旅游业发展水平和整体竞争力。积极发展信息、物流、中介、咨询、金融等现代服务业；改造提升商贸流通、餐饮等传统服务业；发展连锁经营、集中配送、电子商务等现代流通方式；积极发展以居民住宅为重点的房地产和装饰装修业；拓展文化、社区服务等需求潜力大的服务业。

3、坚持“三轮驱动”战略，增创城市建设新优势。切实加强规划建设管理，大力提升省会城市现代化水平，把贵阳建设成建成区面积达到200平方公里，城市人口达到300万人，辐射全省、承东接西、特色突出的区域性中心城市。一是坚持高起点规划。突出“双中心、多组团、众星捧月”的城市空间布局，开展新一轮《贵阳市城市总体规划修编》工作，编制贵阳市区域发展战略规划、贵阳市综合交通规划；基本完成新农村村寨整治规划的编制；加快实施新一轮城镇建设规划，做好贵阳市土地利用总体规划文本的修编和各区、市、县土地总体规划修编工作。二是进一步完善城市配套功能。加强以交通运输、能源水利、公用设施等为重点的城市基础设施建设力度，着力优化和完善城乡基础设施体系。实施“一纵两横三环线”、“一环一横七射线”道路网络体系建设。重点实施城市环线、贵阳至清镇高速公路、金阳至老城区第三条快速通道、金阳新区至百花湖至清镇环湖快速通道、油榨街至小碧高等级公路和城市主干道建设，等级公路建设力争达2000公里以上，启动城市轻轨建设。积极搞好航空港、贵阳铁路枢纽、厦蓉高速公路贵阳段、贵阳至广州高速公路和铁路、贵遵高速公路扎南段等重大项目的协调服务，启动建设修文至金沙海马大桥，建成贵阳绕城高速公路、久铜线贵阳段二级公路、开阳码头港口、息烽至金沙乌江大桥和56个农村客运车站，逐步形成水、陆、空并举的立体交通网络。加强以电力和解决工程性缺水为重点的能源水利建设，重点发展年产30万吨以上煤矿，加快清镇电厂异地搬迁改造，加快建设一批中小型水利工程和污水处理厂、加强煤气气源和储备站点建设。实施城市客运车辆站场建设项目，加大市政基础设施建设。加快再生资源中心、南郊垃圾处置场及全省危险废弃物处置中心项目建设，推进城市污水、垃圾综合利用。三是强力推进金阳新区建设。进一步完善金阳新区封闭运行机制，认真落实好金阳新区鼓励投资扶持政策，支持金阳新区先行发展，引导云岩、白云、乌当加快与金阳新区的连接和融合。坚持高起点规划、高标准建设、高强度投入、高效能管理，加快推进金阳新区产业发展、城乡统筹、一体化建设“三大工程”，努力建设市政公共设施、科教园区、体育文化、行政办公、新型住宅、旅游休闲、现代服务业、高新技术产业“八大城市功能板块”，搭建技术创新共享、孵化、科技中介服务和自主知识产权保护四大创新服务平台，逐步把金阳新区建设成为生态型、数字化、园林式的中心城区，成为我市经济社会发展的重要增长极。四是加快老城区改造提升。重点建设一批枢纽型、功能性、具有较强集散能力的城市基础设施，完善市区交通网络和市区对外交通体系。加强停车场（库）的规划、建设和管理，加快实施“农改超”工程。巩固和扩大南明河三年变清的成果，着力抓好城市排水系统改造和污水处理厂的建设。推进山体公园建设，提升城市绿化档次。建立规范高效的城市管理和城市综合执法体制，在城市精细化管理上下功夫，充分发挥区和街道办事处在城市管理中的积极作用，促进城市管理的法制化和规范化。五是大力推进经济强县（市、区）建设。依托各具特色的资源和区位优势，提升优化经济结构，大力发展特色经济，建成各具优势的产业基地，形成产业布局合理、分工明确、优势互补的县域经济生产力发展布局。大力支持南明区、花溪区、乌当区共同开发龙洞堡、小碧、永乐片区，建设临空经济区，积极支持云岩区“三圈建设”、小河区“三区两带”、乌当区洛湾片区开发、白云区新材料工业园、花溪区清华紫光科技园、清镇市西部工业集中区、修文县铝及铝加工和中药现代化基地建设，鼓励支持息烽县、开阳县发展特色经济，力争各区、市、县生产总值翻一番并全部进入全省经济强县行列。六是加快推进小城镇建设。以交通干线为基础，经济流向为主导，加大城镇基础设施投入力度，增强城镇凝聚力和辐射力，逐步形成由卫星城镇、重点城镇、特色城镇、一般城镇组成的布局合理、设施完善、生态环境优美、特色鲜明的城镇体系，不断提高城市化水平。

4、加快体制机制创新，增创改革开放新优势。一是加大国有企业改革力度。建立健全出资人制度和国有资产授权经营管理制度，落实国有资产经营责任制，完成国有企业改革改制任务和国有大中型企业股份制改革。鼓励和支持非公有制经济进入金融服务、公用事业、基础设施等领域，加强引导和促进非公有制企业管理水平上台阶，大力提升非公有制经济在国民经济中的比重。二是深化财税体制和投融资体制改革。坚持依法治税，应收尽收，不收过头税，坚决制止越权减税和免税，完善和规范税收征管体制。建立公共财政框架和规范的财政转移支付制度，健全政府投资决策责任制度，全面推行政府采购制度。完善项目审批、核准和备案制度。三是深化农村各项改革。全面推进农村综合改革，基本完成乡镇机构和县乡财政管理体制改革任务。加快征地制度改革，探索被征地农民的社会养老保险路子。加快农村合作经营制度改革。改革和创新农村金融体系。四是加大对内对外开放力度。以项目投入为中心，以产业调整为主线，以三个开发区和金阳新区为载体，以珠江三角洲、长江三角洲和环渤海地区及港澳台地区作为招商重点，加强与中、西部经济协作，加大对东盟、日本、韩国及欧美的招商力度。转变对外贸易增长方式，优化出口产品结构，抓紧建好出口基地，创造条件建立出口加工区。进一步加强与东部地区和泛珠三角经济区、成渝经济区和“贵阳城市经济圈”各方沟通与协商，推动区域经济合作和城市经济圈市场一体化。

5、大力推进循环经济和生态经济市建设，增创城市可持续发展新优势。一是大力发展循环经济。抓住列为国家循环经济工作试点城市这一契机，以磷化工、铝加工和煤化工等高耗能行业为突破口，加快循环经济生态工业试点建设，抓好示范项目建设，建设好开阳磷煤化工、清镇煤化工和息烽磷复合肥等生态工业示范基地。继续推进农业科技示范园区和生态经济示范村建设。基本建立企业自觉实施清洁生产的机制和管理体制。二是加快推进生态经济市建设。大力实施“绿色”、“碧水”、“蓝天”、“宁静”四大工程，加强自然保护区和生态示范区的建设和管理，加大石漠化治理、退耕还林还草、天然林保护、长江水源保护、喀斯特生态系统保护和环城、环湖林带等生态工程建设。加强全市水资源的统一规划、利用、节约、保护和管理，实施红枫湖、百花湖、阿哈水库、松柏山水库、花溪水库“一规六环”建设，切实保护好主要饮用水水源。加强工业大气污染的防治，强化汽车尾气、扬尘、饮食油烟和空气污染的防治。加强城市环境噪声的综合整治，实施排放总量控制、排放许可和环境影响评价制度。三是切实加强人口和计划生育工作。继续稳定低生育水平，提高出生人口素质，改善人口结构，引导人口合理分布，积极应对人口老龄化，促进人口与经济、社会、资源、环境协调和可持续发展。

（二）以构建和谐社会为目标，在全省率先实现贵阳社会建设、文化建设新跨越

1、积极推进创新型城市建设，增创自主创新新优势。一是积极推进科技进步和创新。建立完善区域技术创新体系，在优势产业和特色经济领域全面建立产业技术创新支撑平台。建成国家级、省级企业技术中心80家，新建市级工程技术中心100家、国家级重点实验室1至2个、国家级工程技术研究中心3至5个。实施科技创新工程，提高企业技术创新能力，强化企业技术创新的主体地位和企业家的核心作用。整合科技资源，推进合作创新和成果转化。深化科技体制改革，初步建立“结构优化、布局合理、精干高效、纵深配置”的科技创新体系与运行机制。争创国家可持续发展实验区。全面实施“数字贵阳”建设规划，努力提高经济社会信息化水平。二是大力推进开发区加快发展。以增强技术创新能力为核心，加快开发区高新技术产业的聚集发展。建设中国·西部（贵阳）高新技术产业研发基地，把三个开发区建成面向全省的科技创新基地和辐射中心。三是大力推进创新服务体系建设。加强科技创业服务中心建设，建立科技资源共享、科技成果转化、科技企业孵化、科技投融资、技术交易等五大科技中介服务平台。加强技术咨询、技术转让等中介服务。加快发展创业风险投资，鼓励金融机构、民间资本进入科技创新领域。加强知识产权保护，深入开展科普工作。

2、大力提升文化竞争力，增创文化建设新优势。一是大力推进文化体制改革。加快新闻出版、艺术团体、公益性文化事业单位的改革，组建贵阳日报报业集团，推进贵阳电视台、贵阳人民广播电台资源整合，重点扶持有较高艺术水准和能充分体现贵阳地方特色的艺术团体，进一步提高艺术生产及服务水平。二是加大政策扶持力度。市财政每年安排500万元作为市级文化产业发展专项资金；编制并实施2006—2010年市文化事业和文化产业发展总体规划，建立全市文化产业项目库，筹建文化产业园，设立“贵阳市文艺创作奖”，对优秀文艺作品及人才给予奖励。三是大力培植七大文化产业。重点扶持、优先发展现代传媒业、旅游娱乐业、会展文博业、演出培训业、影视传播业、印刷业、民族民间艺术品业等七大文化产业，大力发展文化创意产业，壮大一批龙头文化企业。完善国有文化资产授权经营机制。制定《贵阳文化人才培训学习办法》，大力培养各类文化人才。

3、加快教育事业发展，增创人才队伍建设新优势。一是优先发展教育。继续增加各级财政对义务教育的投入，加强农村学校、薄弱学校和非筑籍子女学校基础设施建设，全面推进义务教育均衡发展。不断提高农村学校和职业教育师资水平。完成县级示范幼儿园和中心幼儿园建设。支持发展民办教育。率先在全省基本普及高中阶段教育。大力发展职业教育，建好贵阳职业技术学院、贵阳护理职业学院和贵阳市职业教育中心，扶持十一个区（县、市）职教中心建设，基本实现高中阶段在校生中普通高中与中等职业教育比例大体相等。加快发展高等教育，使贵阳学院跻身于全省一流大学行列，使全市人均受教育年限达10年以上，进一步抓好现代远程教育和社区教育。二是积极构筑人才高地。大力实施人才强市战略，继续实施“高层次党政人才培养、企业家培养、金筑英才、高技能人才培养、农村实用人才培养”等五大工程，大力培养各类人才。完善优惠政策，积极培养和引进我市重要产业、重点学科、重大项目急需的高层次人才和紧缺人才。加快人才评价向个性化评价和社会化评价机制转变，建立和完善人才评价体系和激励机制。

4、切实解决民生问题，增创社会发展新优势。一是千方百计扩大就业。强化政府促进就业的公共服务职能，深化户籍制度、城镇就业管理制度等改革，健全就业服务体系，加快建立职业技能培训机制，推动劳动预备制度、职业资格证书制度，继续完善就业培训优惠政策，落实对困难群众的就业援助制度，鼓励企业增加就业岗位，形成促进扩大就业的有效机制。二是加快社会保障体系建设。建立健全与我市经济发展水平相适应的社会保障体系，完善城镇职工基本养老、基本医疗、失业、工伤和生育保险制度。推进机关事业单位养老保险制度改革，逐步将进城务工人员纳入城镇社会保障范围。进一步完善和规范农村最低生活、优抚保障机制，完善社会住房保障体系，加强社会保障信息网络建设，积极开展社会救助。三是提高人民群众健康水平。进一步健全突发公共卫生事件应急处理机制、疾病预防控制体系、医疗救治体系、妇幼保健体系、城乡医疗保障体系、卫生执法监督体系和城乡医疗服务体系。加快推进医疗体制改革，完善新型农村合作医疗制度，大力发展城市社区卫生服务，合理配置医疗卫生资源，整顿药品生产和流通秩序，逐步解决群众看病难、看病贵问题。加强体育设施建设，大力开展全民健身运动，提高体育竞技水平。

5、全力打造全省和谐“首善之区”，增创和谐贵阳新优势。一是深入推进“平安贵阳”建设。切实加强社会治安综合治理和社会治安防控体系建设，强化禁毒工作，依法严厉打击各种违法犯罪，整顿和规范市场经济秩序，努力营造“居住在贵阳、创业在贵阳、平安在贵阳”的良好环境。充分发挥信访工作在构建社会主义和谐社会中的基础性作用，完善信访工作责任制，建立完善应急管理体制机制，切实保障公共安全。高度重视安全生产，切实抓好煤矿、交通、消防安全监督管理，整合现有煤矿资源，切实预防和有效减少安全生产事故。加强对各种自然灾害的预测预报和防灾减灾工作。二是加强“文明贵阳”和“诚信贵阳”建设。深入贯彻落实《公民道德建设实施纲要》，全面推进精神文明建设，促进我市经济发展和社会协调进步。加强市场监督，加快建设社会信用体系，建立失信惩戒制度，维护和健全市场秩序，建设“诚信贵阳”。

（三）打造“为民、廉洁、务实、高效”政府，全面加强自身建设

一是提高素质，建设学习型政府。要大兴学习之风，认真贯彻落实《公务员法》，加强公务员培训，树立终身学习、终身接受教育的理念，全面提高行政领导、公务人员的理论素养和执政水平。二是狠抓落实，建设责任型政府。积极倡导说实话、办实事、求实效，坚决克服官僚主义、形式主义作风；深入基层、贴近群众，密切关注并积极处理在经济生活和社会管理中出现的矛盾和问题。三是转变职能，建设服务型政府。要把政府职能转移到经济调节、市场监管、社会管理和公共服务上来。进一步深化行政审批制度改革，规范行政许可事项，清理非行政许可类审批项目，简化审批程序。加强政府创新能力建设，加快电子政务建设，提高办事效率。四是健全机制，建设廉政型政府。严格执行党风廉政建设责任制，建立和完善思想教育、制度规范、监督制度及惩处机制，加大从源头上预防和治理腐败的力度，认真开展治理商业贿赂专项工作，纠正部门和行业的不正之风，加强审计工作，加大审计监督力度，坚决查处各类大案要案，严肃处理违法违纪人员。五是依法行政，建设法治型政府。加强民主法制建设，提高行政立法工作的透明度和公众参与度，重大讨论决定事项要自觉接受市人大及其常委会的法律监督和工作监督，自觉接受各级政协的民主监督，认真听取民主党派、工商联、无党派人士和各人民团体的意见，接受新闻舆论和社会公众监督，促进政府决策的民主化。全面贯彻落实国务院《依法行政实施纲要》，进一步落实行政执法责任制和评议考核制，加强执法监督，认真执行错案追究制度，切实做到依法决策，依法管理，依法办事，营造公正、高效的法制环境。

三、2007年目标任务和工作重点

2007年，是新一届政府的开局之年。全面做好今年的各项工作，对“十一五”规划的全面完成，实现新一届政府制定的各项目标任务具有十分重要的意义。今年经济社会发展的主要预期目标为：全市生产总值增长13%以上，其中，第一产业增加值增长7%；第二产业增加值增长14.5%；第三产业增加值增长12.5%。财政总收入和地方财政收入均增长13%以上。全社会固定资产投资增长18%以上。外贸进出口增长10%。社会消费品零售总额增长14%。城镇居民人均可支配收入和农民人均纯收入均增长8%。万元生产总值综合能耗降低4%，主要污染物排放减少2%。全面完成市委、市政府为民办好的十件实事。

市委、市政府为民办好的十件实事是：（一）免除全市农村和城市低保人群子女义务教育阶段的学杂费和书本费，对在民办学校就读的进城务工农民子女提供义务教育补贴；在农村建设8所标准化学校；改扩建或新建公办中、小学校8所，接纳进城务工农民子女接受义务教育。（二）继续在全市推行新型农村合作医疗制度，农业人口参合率达到90%以上；加强基层卫生服务网络建设，创建10个标准的示范性社区卫生服务中心，建立3个惠民医院，建成金阳医院并投入使用。（三）解决农村15万人饮水安全，新建农村小水池1万口；新建沼气池3.6万口，配套改厕、改圈、改灶3.6万个；完成县、乡、村公路改造1000公里、村寨串户路改造500公里；开工建设10个乡（镇）客车站。（四）新增就业岗位4万个；完成农村富余劳动力非农技能培训2.5万人，有序转移农村富余劳动力就业2万人；新增1000户廉租住房保障，完成经济适用住房建设100万平方米。（五）完成1597个自然村通电视和820个自然村通广播工程；建立四室合一（党员活动室、文化活动室、卫生室、计生室）村级综合楼187个。（六）开工建设贵金线、北京西路，改造朝阳洞路；建成服务大楼立交桥和10座人行过街天桥；开通15条社区公交路线；全市新增停车位3000个。（七）新增公厕20座，新建或改造垃圾中转站13座；开工建设新庄污水处理厂、小河污水处理厂二期及金竹片区排水主干管工程；开工建设全省危险废弃物暨医疗废弃物处置中心。（八）新增绿地30万平方米；创建“绿色社区”20个；建立无公害蔬菜基地150个；在云岩、南明各建一个规范的绿色农贸市场、各建一个农民进城卖菜早菜市场。（九）开工建设金阳新区观山公园和“文化山”社会基础设施项目。（十）市老年大学新教学楼建成投入使用；开工建设市交警指挥中心、市计生宣传技术服务指导中心；改建、重建基层人民法庭6个；解决9个基层派出所办公用房问题。

为完成上述目标，我们要按照中国共产党贵阳市第八次代表大会的要求，立足新起点，增创新优势，推动我市经济社会又好又快的发展，着重抓好以下十个方面的工作：

（一）以推进新农村建设、增加农民收入为着力点，在加强农村基础设施建设、加快现代农业发展上实现新突破

一是加大农村基础设施建设力度。按照“一基三化四提高”的总体要求，大力实施“6543”工程，扎实推进社会主义新农村建设。继续抓好“十村示范、百村先行、千村推进”工程，力争开工建设渔洞峡、金龙水库等亚行贷款水资源管理项目，利用世行贷款开工建设10个乡镇客车站。继续实施万户小水池工程，解决农村15万人饮水安全，抓好水政执法和水资源管理，关停全市公共供水管网覆盖范围内自备水源。继续推进县乡村道路改造1000公里、串户路硬化500公里、基本农田基础设施建设3万亩、中低产田改造5万亩、退耕还林3000亩。新建沼气池3.6万套，积极推进烟水配套工程，实现受益面积2—3万亩。二是加快推进现代农业发展。按照现代农业发展要求，大力推进农业产业化，重点发展贵黄、贵开、贵遵等公路沿线果蔬产业带，续建无公害果树基地1万亩，大力发展蔬菜产业，建立蔬菜产业带40万亩，完成150个无公害蔬菜基地建设，实现蔬菜外销量50万吨。大力扶持花卉产业，新增1800个花卉大棚，建成花卉园艺展示交易中心，力争花卉产值突破2亿元。新建养殖小区200个，重点发展奶牛产业，新增奶牛3600头。积极扶持一批农产品加工企业，支持温氏集团等大型农业龙头企业入住贵阳，建立大型养殖和加工基地，新增3家以上市级龙头企业。三是不断完善农村各项保障制度。全面落实农村各项惠农政策，继续抓好“两免一补”，进一步完善和规范农村最低生活保障制度，逐步提高农村最低生活补助标准，基本实现应保尽保。基本完成第二次农业普查工作。四是加大农技培训和劳务输出工作力度。完成农民实用技术培训35万人（次），完成农村富余劳动力非农技能培训2.5万人，有序转移就业2万人。五是大力发展县域经济。继续坚持“放权、服务、扶持”的原则，尽快研究出台新的市、区利益机制，促进经济强县建设，巩固和提升我市经济强县在全省县域经济发展中的位次。

（二）以加快经济结构调整、转变经济增长方式为着力点，在提升工业经济质量、壮大整体竞争力上实现新突破

一是加快发展支柱产业和特色产业。按照工业强市战略的总体要求，重点支持开磷集团120万吨磷铵、兖矿50万吨合成氨、清镇80万吨氧化铝、清镇配煤基地、中化开磷60万吨硫基复合肥、贵州轮胎公司40万条全钢子午线轮胎生产线等重大建设项目；加快推进清镇电厂异地技改、贵阳卷烟厂异地搬迁、老干妈公司扩建工程；大力扶持益佰、同济堂等制药企业做大做强，举办第三届药博会，确保医药制造业增加值增长30%以上。二是大力发展高新技术产业和培育新兴产业。充分发挥军工企业在技术、装备、人才方面的优势，积极支持贵航集团、振华集团做大做强。抓好贵州斯奈克玛飞机发动机叶片生产基地、西南钛业及关联产业、西南工具厂综合采煤成套系统及压缩机零部件生产线等一批重大建设项目；积极支持推进泛特尔生物制药国家一类新药、龙翔半导体6英寸晶园生产线、威顿晶磷年产3200吨电子级磷酸项目等一批高新技术产业建设力度，确保高新技术产业增加值增长30%以上。三是加快推进园区建设。继续加大高新区金阳科技产业园、贵阳经济技术开发区、新天园区配套设施建设力度，加快高新区孵化器建设，全力推进高新区10平方公里基础设施建设和产业规划，建设好开阳、息烽磷化工产业基地、清镇煤化工和铝工业基地、小河装备及汽车零部件产业基地、白云铝工业和新材料等产业基地，大力推进龙洞堡食品工业园、扎佐医药工业园等建设力度，加快发展各具特色的开发区、工业园区及产业基地。四是大力实施名牌战略。积极支持企业技术创新和信息化建设，帮助企业加强知识产权保护，大力培育和发展具有自主知识产权的名牌产品，力争专利申报量比去年增长20%以上。五是大力促进中小企业及非公有制经济发展。建立完善我市中小企业服务体系；鼓励非公有制经济进入公用事业、社会事业、国有经济结构调整领域，在市场准入、税收政策、土地使用、对外贸易、经济技术合作等方面给予同等待遇，引导支持民营企业不断发展壮大。

（三）以项目带动、促进产业发展壮大为着力点，在优化投资结构、提高投资效益上实现新突破

一是加大基础设施和重大项目的建设力度。加大以交通为主的基础设施建设力度，积极配合做好龙洞堡机场改扩建、贵阳铁路枢纽工程、川黔复线、黔桂线扩建、绕城高速公路、贵遵高速公路扎南段、沪瑞高速公路贵阳至清镇段、清镇至织金公路、贵阳至广州高速铁路、厦蓉高速公路贵阳段等项目建设；开工建设息烽至金沙乌江大桥、开阳至瓮安棉花渡大桥、久铜线麦架至开阳二级公路；积极做好久长至开阳铁路支线建设以及开阳港规划的前期准备工作；加快推进东出口高速公路、贵阳公路主枢纽客货运输站场和10个乡镇车站码头等项目建设。产业化建设方面，重点配合抓好贵州詹阳动力重工有限公司多功能工程车、兰花水泥二期工程、中化开磷年产20万吨磷酸二铵等一批重大建设项目。二是积极筹措建设资金。切实做好项目的筛选和申报工作，积极争取国家、省支持项目的力度。要继续深化亚行、世行、国开行、外国政府贷款项目的前期工作，确保这些项目尽快开工。进一步激活民间资本，扩大民间投资的市场准入范围，形成投资主体多元化。三是切实做好“投转固”工作。加快完成“投转固”工作并实现市场化运作，做到建成一项，转固一项，使项目尽快发挥效益。

（四）以加快商贸流通、努力扩大内需为着力点，在提升现代服务业、促进旅游发展上实现新突破

一是加快市场培育和建设力度。大力实施“双百市场”工程，进一步完善五里冲等大型批发市场配套设施，加快推进浙江商城二期、金阳商贸走廊建设的力度。继续实施“万村千乡”市场工程，着力抓好农贸市场的改造升级和标准化建设。二是加快现代物流业发展。积极引进国内外大型知名物流企业，加快建设二戈寨、扎佐等一批物流基地和配送点，不断提升物流水平和规模，打造成为西南地区重要的物流中心。三是加快培育新的消费热点。积极扩大居民消费，引导和促进居民扩大住房、家用轿车、电信、旅游、文化、教育、体育、休闲娱乐和其他服务性消费。大力发展金融、保险、咨询、社区服务、家政服务、医疗保健服务等成长性好的新兴服务业，提升服务业发展水平。四是加快发展旅游业。以完善旅游基础设施为重点，强力推进青岩古镇、红枫湖景区、乌江峡红色旅游景区、乌当森林温泉度假区、修文野生动物园、阳明文化遗址及开阳南江峡谷喀斯特生态旅游区等景区景点的保护和开发，办好花溪之夏艺术节、兰花博览会、温泉节、红枫湖旅游节、全国汽车拉力锦标赛开阳分站赛等活动，精心打造贵阳“森林之城、避暑之都、休闲胜地”品牌，大力发展绿色旅游、红色旅游、温泉旅游，努力把贵阳建设成为西南乃至全国的重要旅游目的地和服务中心。

（五）以实施“三轮驱动”发展战略、完善城市服务功能为着力点，在加强城市规划、建设和管理、提升城市品位上实现新突破

一是强力推进金阳新区建设。着重抓好清金线、金朱路等一批道路建设，加快推进金阳新区公交枢纽站、客运站、交通信息指挥中心、交警指挥中心建设，完善金阳新区道路交通网络结构，加快金阳新区与云岩、白云、乌当的连接和融合。加快实施“三大工程”和“八大城市功能板块”，全力抓好金阳科教园区、“文化山”、贵阳奥体中心、会展中心、青少年活动中心、科技馆、工人文化宫、妇女儿童活动中心、广电中心、贵阳日报印务发行中心、互联网交换中心、档案馆、图书音像城、贵阳护理职业学院等一批项目。全力配合贵州师大、省电大等一批院校进驻金阳，开工建设观山公园、加快推进石林公园等旅游休闲风景区的建设。大力支持新世界地产、世纪金源地产、金元国际新城等一批房开项目，加快推进金阳新区高新技术产业发展，不断完善金阳城市服务功能，加快产业和人气聚集。二是大力加强老城区人居环境建设。以创建全国文明城市、国家园林城市、国家卫生城市、国家环境保护模范城市为目标，本着老城区“稀化、优化”的原则，切实加强城市规划、建设和管理。启动我市区域发展战略规划、综合交通规划编制工作；做好我市城市总体规划修编。加强城市基础设施建设，加快再生资源交易中心、南郊垃圾填埋场及全省危险废弃物处置中心等项目建设；加快推进贵金线（瑞金北路延伸段）、水东线、油小线、西南环线、市北路二期、北京西路（北京路至金阳）等道路建设；实施朝阳洞路改造工程；做好将富水路改造成为商业步行街的前期准备工作；抓好新庄污水处理厂建设、完成小河污水处理厂二期主体工程、金竹片区排水主干管、陈亮河综合整治等项目建设。加快三桥马王庙片区开发建设。积极做好城市轻轨交通建设规划的编制和申报工作，抓好贵阳电子地名信息系统建设，加快停车站（场）建设，建设10座人行过街系统，改造大营坡、紫林庵、蟠桃宫等3个交叉口环岛，优先发展公交，积极推行公交进社区，提高城市交通通行能力。深入推进城区“城中村”改造，完成云岩、南明拆除违章建筑100万平方米。加强建筑市场、燃气市场、房地产市场的监管工作，抓好煤气气源和储备站点建设，保持贵阳市房价总体稳定。建成经济适用住房100万平方米，新增廉租住房保障1000户。三是加快小城镇建设。抓好新一轮城镇总体规划修编，加快重点城镇供水、排水、道路等基础设施建设，以产业发展为核心，完善城镇配套功能，逐步形成各具特色的卫星城镇、中心城镇。四是切实强化城市管理。加强城市精细化、人性化管理，着力打造精品，建设魅力贵阳。加大城市管理综合执法力度，深入开展“整脏治乱”活动。加强对城市公用设施的维护和管理。加大对违法建筑、乱停乱放、占道经营等行为的查处力度，突出抓好户外广告、门头牌匾的整治工作，着力整治小街小巷等卫生死角，强化夜间城市管理，对延安路、神奇路占道经营的夜市摊点进行综合整治。云岩、南明各建一个农民进城卖菜早菜市场。

（六）以深化改革、营造公平市场环境为着力点，在推进体制机制创新、完善市场经济体制上实现新突破

一是进一步深化国资管理体制改革和国企改革。积极推行国有企业负责人经营业绩考核制，建立起“归属清晰、权责明确、保护严格、流转顺畅”的现代产权制度，盘活存量资产，引进战略合作伙伴投资对国有企业进行股份制改革，基本完成市属国企改革重组任务。积极扶持一批企业上市。二是继续深化农村各项改革。加快推进农村义务教育体制改革、县乡财政体制改革、乡镇机构改革；继续深化农村信用社、粮食流通体制、供销社、水利产权制度等方面的改革。三是深化行政管理体制改革。继续完善行政事务管理、行政审批管理等各项改革，稳步推进事业单位改革，强化社会管理和公共服务职能，提高行政效率和服务水平。四是强化投融资和财税体制改革。要充分利用银行、证券资本市场等进行融资，协调好与各金融机构的关系，大力支持贵阳商业银行做强做大，建立运作良好的融资和偿贷机制，不断提高我市融资水平。积极推行“代建制”，建立投资决策制，实行政府投资项目公示制度和重大投资项目评估制度。进一步落实项目法人制、招投标制、工程监理制和审计验收决算制，防止腐败现象的发生。继续深化部门预算管理、国库集中支付、政府采购、收支两条线和政府收支分类改革为核心的财政改革，加强税源建设，努力涵养税源。加大对政府非税收入和行政事业性资产的管理力度，进一步压缩财政一般性开支，坚决杜绝随意新开增支口子，提高财政资金的使用效益。

（七）以大力实施“环境立市”、营造良好的投资环境为着力点，在扩大对内对外开放、提高招商引资水平和质量上实现新突破

一是切实抓好项目库建设。根据国家投资方向，结合我市产业发展实际，精心策划包装1000个市场前景好、带动作用强的大项目、好项目，改造提升我市传统制造业和高新技术企业水平。二是进一步改善投资环境。要用好、用足、用活各项优惠政策，把贵阳政务服务中心搬迁到金阳，并建设成全省一流的政务服务中心，着力营造公正、公平、高效的法制环境、政策环境、服务环境。三是努力提高对外开放水平。要紧紧抓住《外交部支持贵州对外开放有关措施》出台的机遇，积极开展对外交流与合作，完善外贸服务体系，创新引资方式，不断提高利用外资的质量和水平。四是积极扩大招商引资规模和领域。大力推进贵阳城市经济圈建设，积极做好赴长江三角洲、珠江三角洲、环渤海经济圈、新加坡、韩国举办大型投资、旅游推介活动的各项工作。抓住第四届泛珠三角区域经济协作会、黄果树瀑布节等一系列经贸活动的契机，不断加大项目推介力度，大力吸引对我市产业升级具有重大带动作用的项目和知名企业，重点引入500万美元以上的外资项目和5000万元以上的项目，力争引进2家以上世界500强企业。

（八）以生态建设和保护、大力发展循环经济为着力点，在推进生态经济市、构建资源节约型和环境友好型社会上实现新突破

一是加强生态环境建设和保护。加强环境治理和保护，大力实施石漠化治理、退耕还林还草、天然林保护、长江水源保护、喀斯特生态系统保护和环城、环湖林带、城市绿化等生态工程建设，抓好矿产资源开发秩序的全面整顿和规范工作，加大地质灾害防治力度。二是加大污染防治力度。加强水污染防治，重点抓好红枫湖、百花湖和阿哈水库等饮用水源保护。严格控制各类大气污染物的排放，进一步改善城市空气质量。加强城市噪声综合整治，营造良好的城市环境。三是深入开展资源节约与综合利用。加强节能、节水、节材、节地，扶持发展污染小、消耗低、效益高的资源节约型产业。加大对重点产品、重点企业的技术改造力度，做好对全市15户省重点节能企业的监测管理工作，大力推行新型建筑材料，切实抓好资源综合利用工作。四是大力发展循环经济。大力推进循环经济试点项目，继续全面推进开阳磷煤化工、清镇铝煤化工、息烽磷化工示范基地、修文扎佐医药园区以及乌当生态农业示范园等循环经济项目建设；大力推进小油桐生产柴油产业化，抓好“湿法磷酸节能降耗工艺”等重大课题的研究，继续推进再生资源绿色回收利用体系建设。

（九）以推进创新型城市、繁荣社会主义文化为着力点，在促进经济社会协调发展、推进社会事业全面进步上实现新突破

一是大力实施科教兴市和人才强市战略。积极推进科技进步和技术创新。完成重大科技项目100项，科技成果鉴定50项。不断深化科技体制改革，建立健全科技风险投资机制，加强以企业为主体的技术创新体系建设，加速科技成果转化，抓好科技创新平台建设，建设中国西部（贵阳）高新技术产业研发基地，启动创新型企业试点工作，新建市级企业技术中心15家，完成贵阳药用资源博物馆二期等一批科普项目建设。大力推进各类人才培育工程，努力创新人事人才工作机制，加强人才市场体系建设。二是优先发展教育事业。进一步巩固和提高九年制义务教育水平，全面实施素质教育，合理配置教育资源，促进教育公平。抓好全市中小学布局调整规划，稳步推进农村中小学标准化建设，继续抓好国家“两免一补”政策的落实，改造城区薄弱初中学校，改扩建8所公办学校，积极接纳外来务工子女接受公办义务教育。大力发展职业教育，继续发展成人教育、幼儿教育，构筑终身教育体系。抓好开阳、清镇、乌当、白云县级职教中心建设，积极争取实验三中、民中、息烽中学、修文中学进入省级示范性高中行列。迎接全国“两基”工作督导检查，组建贵阳护理职业学院、贵阳职业技术学院，办好贵阳学院。三是大力发展文化和体育事业。深化文化体制改革，积极挖掘和保护民族民间文化资源，抓好贵阳大剧院、贵阳美术馆的管理使用和县乡文化馆、社区文化活动中心建设，加快贵阳数字内容产业园产业的聚集发展，举办好亚洲青年动漫大赛及颁奖典礼和第六届中国舞蹈“荷花奖”民族民间舞蹈大赛。全面完成全市县级“两馆”建设；完成全国重点文物保护单位规划，抓好三元宫的环境整治和文昌阁的修缮工作。切实加强民族宗教工作，抓好仙人洞、清真寺的修缮工作。积极推进体育事业发展，力争开工建设贵阳奥体中心。大力开展全民健身运动，抓好贵阳市国民体质监测体系工作。四是加快广电和信息化建设。加快有线网络数字化进程，做好文化信息资源共享工程，继续推进城镇有线电视用户的数字化整体转换。继续抓好“村村通”工程。积极推进“一站两网四库”和“金盾”、“金土”等金字系列工程建设，实施好“数字金阳”信息化工程，继续推进贵阳市地理信息系统二期工程和企业信息化“1261”工程建设。五是积极发展卫生事业。继续推行新型农村合作医疗制度，全市农民参合率达90%以上。加强农村卫生基础设施建设，改扩建乡（镇）卫生院8个，建设村级综合楼187个，加强农村食品卫生安全监督，做好艾滋病、结核病、伤寒副伤寒病等重大疾病和人畜（禽）共患疾病防控工作。大力发展社区卫生服务，进一步健全新型城市医疗卫生服务体系，逐步解决群众“看病难、看病贵”的问题。六是加强人口与计划生育工作。按照“依法管理、村（居）民自治、优质服务、政策推动、综合治理”的要求，继续稳定低生育水平，提高出生人口素质。继续深入开展关爱女孩行动，努力遏制出生人口性别比偏高势头。扎实抓好流动人口计划生育服务和管理长效工作机制的落实。切实兑现对计生家庭的“奖、扶、医、帮、优”等系列优惠政策，实现少生与优生、少生与快富、少生与健康、少生与保障相结合。在全省率先建立人口计生工作新机制，全面提升人口计生工作整体水平。

（十）以解决关系人民群众切身利益的问题、加强社会管理为着力点，在维护社会稳定、构建和谐贵阳上实现新突破

一是大力促进就业和再就业。切实落实促进就业再就业的一系列政策，加大劳动力市场的建设培育力度，形成促进就业的良性体制和机制，在全市范围内开展“零就业”家庭就业援助及充分就业社区试点工作，使全市城镇登记失业率控制在4.5%以内。二是切实加强和完善社会保障体系建设。继续扩大基本养老、基本医疗、失业、工伤、生育保险覆盖面，进一步完善城镇居民最低生活保障制度；加快农村社会保障制度建设，逐步完善农村居民最低生活保障制度和被征地农民基本生活保障制度；做好为两城区80岁以上贫困、残疾、鳏寡孤独老人免费提供家政服务的试点工作；切实解决低收入弱势群体的生活、就医及其子女入学等问题，关注妇女儿童、老年人、残疾人的社会保障工作。三是强化安全生产。加强应急机制建设，重点抓好食品药品安全，狠抓矿山、交通、建筑施工等各项安全工作，严防各类重特大恶性事故的发生，努力维护人民群众合法权益和社会公共安全。四是扎实推进平安贵阳建设。加强社会治安综合治理和治安防控体系建设，认真开展黄、赌、毒专项治理，依法严厉打击各种刑事犯罪。建立刑事技术中心。认真做好人民群众来信来访工作，抓好矛盾纠纷排查责任制的落实，坚决纠正各种侵害群众利益的行为，切实维护国家安全和社会稳定。五是大力推进精神文明建设和民主法制建设。以“争创全国文明城市、构建和谐贵阳”为目标，深入开展文明城市创建活动，继续认真贯彻《公民道德建设实施纲要》，积极推动“诚信贵阳”建设，不断提高社会文明程度和城乡文明水平。广泛开展道德实践活动，大力弘扬民族精神和时代精神，形成与社会主义市场经济相适应的思想道德体系。加强未成年人思想道德建设，促进青少年健康成长、全面发展。加强拥军优属、拥政爱民工作，确保实现第五次争创全省和全国双拥模范城市的目标。认真贯彻落实国务院依法行政实施纲要，大力推进依法治市规划的实施，完善基层民主管理制度，进一步推行政务公开、厂务公开、村务公开。认真办好人大代表议案、建议和政协委员提案，加强政府部门与各民主党派的联系。深入开展普法宣传教育，做好法律服务和法律援助工作，大力推进社会管理法制化和行政执法规范化。

各位代表！前不久结束的中国共产党贵阳市第八次代表大会描绘了贵阳未来五年的发展蓝图和美好前景，新的目标鼓舞人心，新的形势催人奋进，新的事业任重道远。让我们紧密团结在以胡锦涛同志为总书记的党中央周围，高举邓小平理论和“三个代表”重要思想伟大旗帜，认真贯彻落实党的十六届六中全会精神，在省委、省政府和市委的领导下，在市人大、市政协的监督、支持下，紧紧依靠全市人民，只争朝夕、奋发进取、攻坚克难、扎实工作，努力开创贵阳更加美好的未来，使贵阳带动辐射能力更强、城乡环境更美，人民生活更富足，社会更和谐，实现贵阳经济社会发展的历史性跨越！
